# Prediction of left atrial appendage dysfunction by atrial strain imaging in patients with rheumatic mitral stenosis in sinus rhythm

**DOI:** 10.1093/ehjopen/oeag130

**Published:** 2026-08-20

**Authors:** Jamal Yusuf, Gaurav Sharma, Ankit Bansal, Vimal Mehta, Ankur Gautam, Lovedeep Singh, Mohit D Gupta, Rajat Chaudhary, Vishal Batra, Girish M Palleda, Safal Safal, Arima Nigam, Sumod Kurian, Partho P Sengupta

**Affiliations:** Department of Cardiology, Govind Ballabh Pant Institute of Postgraduate Medical Education & Research, Room No. 127, First Floor, Academic Block, New Delhi, Delhi 110002, India; Department of Cardiology, Govind Ballabh Pant Institute of Postgraduate Medical Education & Research, Room No. 127, First Floor, Academic Block, New Delhi, Delhi 110002, India; Department of Cardiology, Govind Ballabh Pant Institute of Postgraduate Medical Education & Research, Room No. 127, First Floor, Academic Block, New Delhi, Delhi 110002, India; Department of Cardiology, Govind Ballabh Pant Institute of Postgraduate Medical Education & Research, Room No. 127, First Floor, Academic Block, New Delhi, Delhi 110002, India; Department of Cardiology, Govind Ballabh Pant Institute of Postgraduate Medical Education & Research, Room No. 127, First Floor, Academic Block, New Delhi, Delhi 110002, India; Department of Cardiology, Govind Ballabh Pant Institute of Postgraduate Medical Education & Research, Room No. 127, First Floor, Academic Block, New Delhi, Delhi 110002, India; Department of Cardiology, Govind Ballabh Pant Institute of Postgraduate Medical Education & Research, Room No. 127, First Floor, Academic Block, New Delhi, Delhi 110002, India; Department of Cardiology, Govind Ballabh Pant Institute of Postgraduate Medical Education & Research, Room No. 127, First Floor, Academic Block, New Delhi, Delhi 110002, India; Department of Cardiology, Govind Ballabh Pant Institute of Postgraduate Medical Education & Research, Room No. 127, First Floor, Academic Block, New Delhi, Delhi 110002, India; Department of Cardiology, Govind Ballabh Pant Institute of Postgraduate Medical Education & Research, Room No. 127, First Floor, Academic Block, New Delhi, Delhi 110002, India; Department of Cardiology, Govind Ballabh Pant Institute of Postgraduate Medical Education & Research, Room No. 127, First Floor, Academic Block, New Delhi, Delhi 110002, India; Department of Cardiology, Govind Ballabh Pant Institute of Postgraduate Medical Education & Research, Room No. 127, First Floor, Academic Block, New Delhi, Delhi 110002, India; Department of Cardiology, Govind Ballabh Pant Institute of Postgraduate Medical Education & Research, Room No. 127, First Floor, Academic Block, New Delhi, Delhi 110002, India; Department of Cardiovascular Medicine, Mayo Clinic Health System, Mayo Clinic, 200 First Street SW, Rochestser, MNRochestser, MN 55905, USA

**Keywords:** Mitral stenosis, Sinus rhythm, Left atrial strain, Left atrial appendage

## Abstract

**Aims:**

In severe rheumatic mitral stenosis (MS) with sinus rhythm, left atrial appendage (LAA) dysfunction increases thromboembolic risk, yet clinical guidelines provide no recommendations, and its assessment via transoesophageal echocardiography is not feasible for routine or repeated use. This study assessed whether left atrial (LA) strain imaging could non-invasively predict LAA contractile dysfunction.

**Methods and results:**

We prospectively enrolled 138 patients with severe rheumatic MS in sinus rhythm who underwent transthoracic and transoesophageal echocardiography. Left atrial appendage inactivity was defined as LAA emptying velocity < 25 cm/s. Left atrial reservoir strain (LASr), conduit, and contractile strain were quantified using speckle-tracking echocardiography. Multivariable logistic regression and receiver operating characteristic analyses were performed to evaluate predictors of LAA inactivity. Left atrial appendage inactivity was observed in 108 patients (78.2%), and LAA thrombus was detected in eight patients (5.8%)—all in sinus rhythm. In multivariate analysis, LASr was the strongest independent predictor of LAA inactivity [odds ratio: 0.61 per 1% increase; 95% confidence interval (CI): 0.50–0.75; *P* < 0.0001]. Left atrial reservoir strain yielded an area under the ROC curve of 0.950 (95% CI: 0.911–0.989), with a threshold of ≤22.95% showing a sensitivity of 93.5% and specificity of 90% for predicting LAA inactivity. Impaired LA strain identified patients with elevated fibrinogen, D-dimer, and the presence of LAA thrombus.

**Conclusion:**

Left atrial strain imaging provides a robust non-invasive predictor of LAA dysfunction in severe rheumatic MS patients in sinus rhythm. These findings support the potential clinical utility of LA strain in improving risk stratification and guiding anticoagulation decisions in this under-recognized high-risk population.

## Graphical Abstract

**Graphical Abstract:**
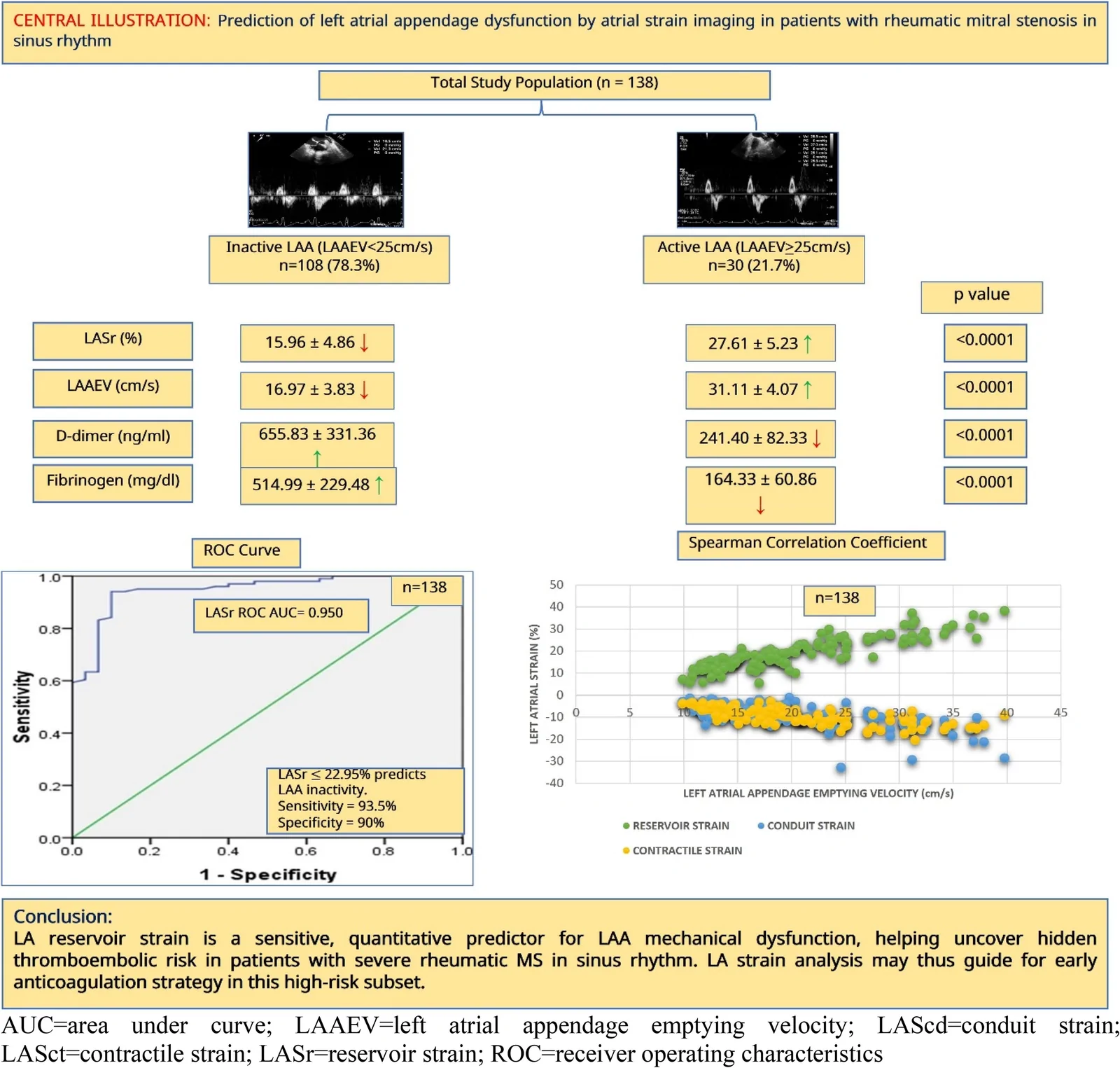

## Introduction

Systemic thromboembolism remains a major source of morbidity and mortality in patients with rheumatic mitral stenosis (MS).^1^ Among these patients, the left atrial appendage (LAA) is the most common site of thrombus formation, with LAA thrombus reported in 6.6–13.5% of patients—even in the presence of sinus rhythm (SR).^2–5^ Studies have consistently shown that thrombus formation in this population is closely linked to LAA inactivity, characterized by reduced contractility and stasis leading to left atrial spontaneous echo contrast.^5–12^

In patients with severe MS (mitral valve area ≤ 1.5 cm^2^), impaired LAA contractile function—commonly defined as LAA emptying velocity (LAAEV) < 25 cm/s—is frequently observed.^4^ However, direct evaluation of LAA function requires transoesophageal echocardiography (TEE), a semi-invasive procedure that is not always feasible for routine or repeated assessment due to patient tolerance and procedural risks.^13–15^

Current guideline-based recommendations address anticoagulation in patients with rheumatic MS and atrial fibrillation (AF),^16–18^ but no guidance exists for patients in SR with inactive LAA, despite mounting evidence that this subgroup carries a significant thromboembolic risk. This represents a critical gap in risk stratification and management.

Given that global left atrial (LA) mechanical dysfunction is closely coupled with impaired LAA function,^19–21^ we hypothesized that LA strain imaging could serve as a non-invasive tool to predict LAA inactivity in patients with severe rheumatic MS in SR.

## Methods

This was a prospective single-centre cross-sectional observational study conducted from July 2023 to June 2024 at the Govind Ballabh Pant Institute of Postgraduate Medical Education and Research (GIPMER) and Maulana Azad Medical College (MAMC), New Delhi, after approval of the Institutional Ethics Committee on Clinical Investigation (Approval no.F.1/IEC/MAMC/111/04/2024/No.487). All patients provided written informed consent. The primary endpoint was the presence of LAA inactivity in patients with severe rheumatic MS in SR. The secondary endpoints were the presence of elevated coagulation parameters, serum fibrinogen and D-dimer levels, and the presence of LAA thrombus.

Study group included men and women aged ≥18 years with severe rheumatic MS in SR (MVA ≤ 1.5 cm^2^ by planimetry).^16^ Patients were excluded from the study if they had paroxysmal AF on 24-h Holter monitoring, coexisting left ventricular dysfunction (ejection fraction < 50%), congenital heart disease, or any contraindication for TEE. Patients were also excluded if they had diabetes mellitus, hypertension, renal or hepatic dysfunction, overt malignancy, pregnancy, chronic inflammatory diseases such as systemic lupus erythematosus, rheumatic arthritis, human immunodeficiency virus infection, or a history of systemic or pulmonary embolism.

### Echocardiography

All participants underwent comprehensive transthoracic echocardiography (TTE) using a Philips EPIQ 7C system (Philips Medical Systems, Andover, MA, USA) equipped with a 1–5 MHz transducer. Standard two-dimensional and Doppler imaging were obtained in parasternal long- and short-axis and apical two- and four-chamber views following American Society of Echocardiography (ASE) guidelines.^22^ Left atrial volume was calculated using the biplane Simpson method and indexed to body surface area. Tissue Doppler imaging was recorded at both septal and lateral mitral annuli.

Transoesophageal echocardiography was performed using a 5-MHz X7-2t probe (Philips EPIQ 7C system, Philips Medical Systems, Andover, MA, USA) in mid-oesophageal two- and four-chamber views. The LAA flow velocities were measured using pulsed-wave Doppler with the sample volume positioned 1 cm below the LAA orifice, ensuring minimal wall artefact interference.

**Figure 1:**
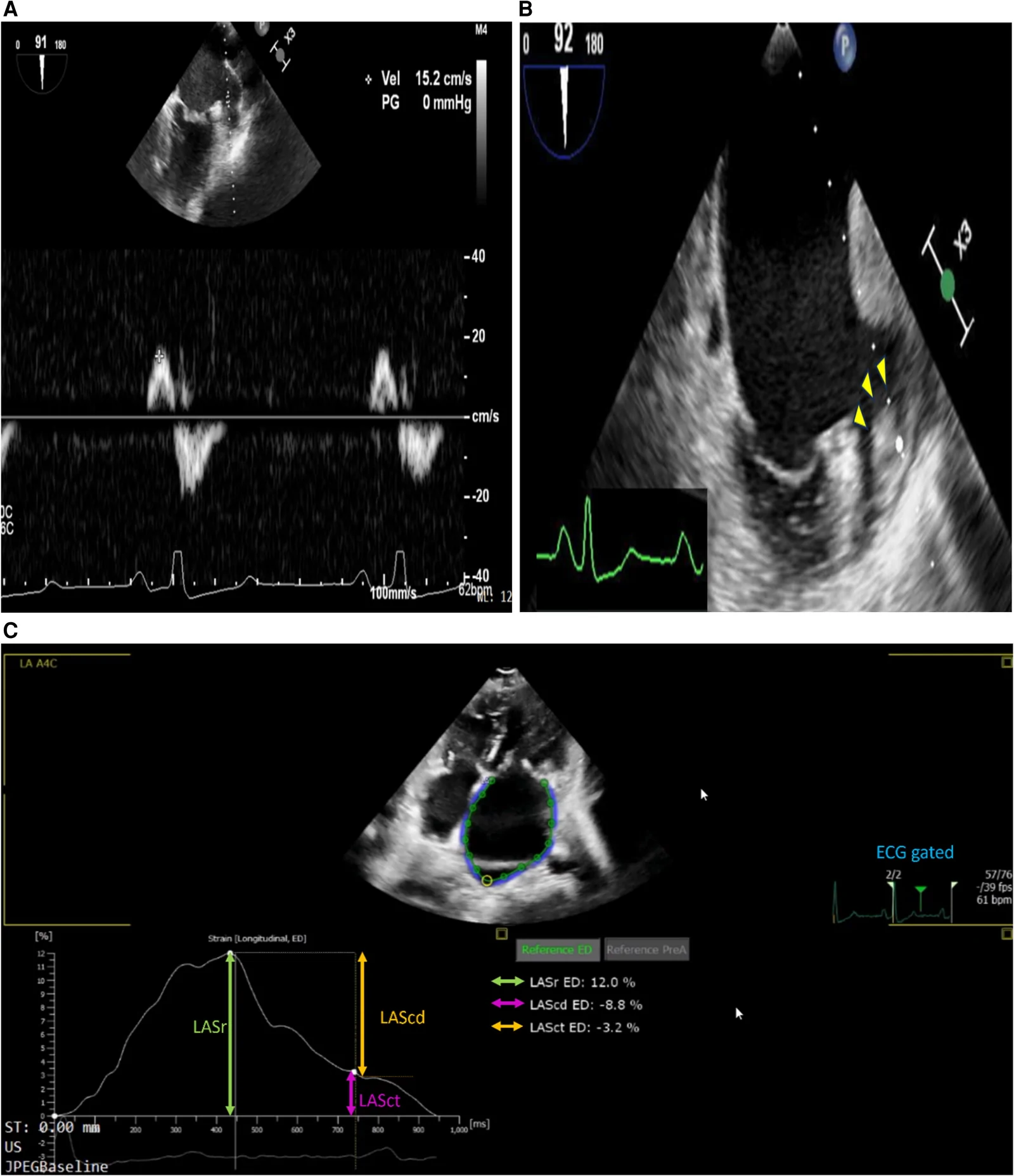
The pulse wave Doppler on TEE showing LAAEV (+). Pulse-wave Doppler tracing from the left atrial appendage (LAA) shows the positive wave that follows the P-wave on the ECG, representing LAA emptying, showing its contractile function (*A*). Transoesophageal echocardiography mid-oesophageal two-chamber view (ME-AV SAX view) showing LAA thrombus (*B*). The image highlights typical features of rheumatic mitral stenosis related atrial stasis, with thrombogenic LAA and absent appendage contraction consistent with mechanical inactivity. The strain curve shows two distinct peaks: the first positive peak before mitral valve opening represents LASr, while the second negative deflection before atrial contraction indicates LASct. The difference between these two peaks defines LAScd (*C*).

- LAAEV: positive flow after the P-wave, representing contractile function.
- Inactive LAA was defined as LAAEV < 25 cm/s.^4,23^ Each velocity was averaged over three consecutive cardiac cycles in two views (*Figure 1A*). Left atrial spontaneous echo contrast was graded 0–4+ according to Fatkin’s criteria^24^ (*Figure 1B*).

### Speckle-tracking echocardiography

One lead electrocardiogram was recorded continuously during the speckled tracking by TTE using a Philips EPIQ 7C fitted with a commercially available S1–5 MHz transducer as per guidelines.^25^ Speckle-tracking analysis was performed on TTE images acquired at 60 ± 10 frames/s using Auto-Strain QLAB 13.0 software (Philips Medical Systems). The LA endocardial borders were traced in the apical four-chamber view, using the QRS onset as the reference. Reservoir (LASr), conduit (LAScd), and contractile (LASct) strains were automatically computed. Tracking quality was visually verified, and manual corrections were applied when necessary. The LA strain curves with two peaks were generated as shown in *Figure 1C*.

- The first (positive) peak before mitral valve opening represented *LASr*
- The second (negative) peak before atrial contraction represented *LASct*
- Their difference defined *LAScd*

All echocardiograms were interpreted independently by two blinded observers, with discrepancies resolved by consensus.

No artificial intelligence (AI)–assisted technologies (such as large language models, chatbots, or image creators) were used in the production of submitted work.

### Haematological investigations

Venous blood samples were drawn without venous stasis into trisodium citrate tubes, centrifuged at 3000 × *g* for 15 min, and plasma stored at −70°C. Fibrinogen and D-dimer levels were quantified using immunoturbidimetric assays (Elite Pro, Roche Cobas C501, Mannheim, Germany) by blinded biochemists.

### Statistical analysis

The sample size calculation was based on previously published study on prevalence and predictors of LAA inactivity in patients with rheumatic MS.^7^ Using a single-proportion formula with 95% confidence level and an absolute precision of 10%, the minimum sample size required was calculated to be 76 patients. To account for potential dropouts and non-interpretable echocardiographic studies and to enable subgroup and secondary analyses, a final sample size of 100 patients was considered appropriate. To account for exclusions, ensure robustness of secondary analyses, and enhance the precision of strain cut-off estimation and subgroup comparisons, consecutive eligible patients were enrolled during the study period, resulting in a final sample size of 138 patients. Thus, for the present study, we recruited 138 patients to reduce the margins of error. The data were analysed using the statistical software SPSS version 25 (IBM Corp., Armonk, NY, USA). The mean and standard deviation, or median and 25th to 75th percentile, were reported for the continuous variables and the number and percentage for the categorical variable. The normality was assessed using the Shapiro–Wilk test. The variable violating the normality condition was reported with a median and 25th to 75th percentile and compared between the active LAA group (LAAEV ≥ 25 cm/s) and inactive LAA group (LAAEV < 25 cm/s) using the Mann–Whitney *U* test. The variable that fulfilled the normality condition was reported with mean and standard deviation, and an unpaired Student’s *t*-test was performed to compare the two groups.

The receiver operating characteristic (ROC) curve was applied to find the discriminant ability of the reservoir, conduit, and contractile strain. The optimal cut-off was obtained using the Youden index. The Spearman correlation was applied to find the strength of association among these variables. A *P*-value of <0.05 was considered significant. Logistic regression determined independent predictors of LAA inactivity. Three separate models were built due to multicollinearity in the reservoir, conduit, and contractile strain %. Model calibration was tested with the Hosmer–Lemeshow test. The discriminative ability of each model was assessed by the area under the ROC curve (AUC) with 95% confidence intervals (CIs), and overall, correct classification with sensitivity and specificity at a probability cut-off point of 0.5 was determined. The discriminant analysis was also performed to check the robustness of the logistic model. In addition, ROC curves of LASr were compared with conventional parameters (LAVI, MVMG, and MVA) to evaluate incremental diagnostic value. Inter-observer variability was assessed using intraclass correlation coefficients (ICC). A sensitivity analysis was performed using a stricter definition of LAA inactivity (LAAEV ≤ 18 cm/s) to evaluate the robustness of the model.

## Results

### Patient’s characteristics

A total of 140 patients with severe rheumatic MS in SR underwent TEE and TTE after meeting the inclusion criteria. Two patients had paroxysmal AF on Holter monitoring; hence, they were excluded from the study, and the remaining 138 patients (mean age: 36 ± 9 years, and 78% females) were enrolled in the final analysis. Clinically, 73.9% (*n* = 102) of patients were in New York Heart Association (NYHA) class II, 19.6% (*n* = 27) in NYHA class III, and 6.5% (9) in class IV (*Table 1*).

**Table 1:** Comparison of baseline demographic, haematological, and echocardiographic parameters of group a and group B patients

Variable	Group A LAAEV (<25 cm/s) *n* = 108	Group B LAAEV (≥25 cm/s) *n* = 30	*P*-value
**Age, years**	35.11 ± 8.03	38.20 ± 11.43	0.173
**Sex, female**	84 (77.7%)	23 (76.6%)	0.200
**Duration, months**	24 [15–36]	21 [12–60]	0.910
**BMI, kg/m^2^**	23.74 ± 3.36	24.28 ± 2.65	0.286
**AHR, 24-h Holter**	79.45 ± 8.37	76.30 ± 6.84	0.070
**HR TTE, per min**	78.30 ± 15.00	73.83 ± 14.39	0.143
**HR TEE, per min**	98.01 ± 22.00	93.47 ± 18.41	0.258
**Haemoglobin, g/dL**	11.91 ± 1.51	12.58 ± 1.80	0.071
**Platelets count, ×10^3^/mm^3^**	1.97 ± 0.81	2.06 ± 0.92	0.603
**Total bilirubin, mg/dL**	0.96 ± 0.27	0.63 ± 0.24	0.109
**SGOT, IU/L**	24.67 ± 7.71	27.66 ± 11.77	0.102
**SGPT, IU/L**	22.83 ± 13.50	23.43 ± 16.69	0.857
**Total protein, g/dL**	7.43 ± 0.90	7.61 ± 0.60	0.216
**Blood urea, mg/dL**	25.96 ± 11.34	26.57 ± 9.95	0.775
**Creatinine, mg/dL**	0.79 ± 0.18	0.85 ± 0.21	0.107
**ESR, mm/h**	19.85 ± 11.07	18.43 ± 10.22	0.512
**CRP, mg/dL, median (IQR)**	1.44 [0.76–1.59]	1.60 [1.10–2.670]	0.198
**Bleeding time, s**	77.24 ± 14.27	71.00 ± 10.22	0.357
**Clotting time, s**	596.12 ± 58.14	602.17 ± 87.44	0.514
**INR**	1.0 [0.8–1.3]	0.9 [0.9–1.0]	0.960
**aPTT, s**	29.21 + 3.84	28.07 + 3.88	0.760
**Fibrinogen (mg/dL)**	514.99 ± 229.48	164.33 ± 60.86	<0.0001
**D-dimer (ng/mL)**	655.83 ± 331.36	241.40 ± 82.33	<0.0001

Values are mean ± standard deviation, or median [25th–75th percentile, *n* (%)], unless otherwise indicated.

AHR, average heart rate measured by 24-h Holter; aPTT, activated plasma thromboplastin time; CRP, C-reactive protein; ESR, erythrocyte sedimentation rate; INR, international normalized ratio; SGOT, serum glutamic-oxaloacetate transaminase; SGPT, serum glutamic pyruvate transaminase, IQR, interquartile range.

Based on LAAEV, patients were divided into two groups: group A with inactive LAA (LAAEV < 25 cm/s) and group B without LAA inactivity (LAAEV > 25 cm/s). There were 108 patients (78.2%) who had inactive LAA (group A), and the remaining 30 patients (21.8%) had normal LAA contractile function (group B). There was no significant difference in age, sex distribution, body mass index, and duration of symptoms between the two groups (*Table 1*). Serum fibrinogen and D-dimer were significantly higher in group A compared to group B (514.99 ± 229.48 vs. 164.33 ± 60.86 mg/dL; *P* < 0.0001) and (655.83 ± 331.36 vs. 241.40 ± 82.33 ng/mL; *P* < 0.0001), respectively (*Table 1*).

### Standard echocardiographic parameters

There was a significant difference in mean gradient across the mitral valve (*P* = 0.016) and left atrial volume index (LAVI; *P* = 0.045) between the two groups. The mean LAAEV in group A was significantly lower in comparison to group B (16.97 ± 3.83 cm/s vs. 31.11 ± 4.07 cm/s; *P* < 0.0001). A significantly higher number of patients in group A had dense smoke (grade 2+ to 4+) than in group B [100 (92.6%) vs. 6 (20%); *P* < 0.001], and LASr, LAScd, and LASct were significantly lower in group A (*P* < 0.001; *Table 2*).

**Table 2:** Comparison of standard echocardiographic parameters of group a and group B patients

Variable	Group A LAAEV (<25 cm/s) *n* = 108	Group B LAAEV (≥25 cm/s) *n* = 30	*P*-value
**Transthoracic echocardiographic parameters**			
** Wilkin’s** s**core**	8.0 (8.0–9.0)	8.0 (7.0–8.0)	0.054
** MVA**, **planimetry, cm^2^**	0.99 ± 0.23	1.07 ± 0.21	0.077
**MVMG, mmHg**	13.52 ± 6.41	10.33 ± 6.13	0.016
**LAVI, mL/m^2^**	63.17 ± 27.23	52.73 ± 21.81	0.045
**LVEDV, mL**	82.99 ± 24.50	85.36 ± 28.52	0.680
**LVESV, mL**	30.18 ± 12.81	35.57 ± 15.42	0.087
**LVEF, %**	59.18 ± 7.89	59.69 ± 7.34	0.742
**TAPSE, mm**	20.3 ± 4.7	20.6 ± 3.8	0.743
**2-D transoesophageal echocardiographic parameters**			
**LAAEV, cm/s**	16.97 ± 3.83	31.11 ± 4.07	<0.0001
**LASEC grade**			
**0 or 1+**	8 (7.4%)	24 (80%)	<0.0001
**2+**	34 (31.5%)	6 (20%)	
**3+**	47 (43.5%)	0 (0%)	
**4+**	19 (17.6%)	0 (0%)	
**LAA thrombus**	8 (7.4%)	0 (0%)	
**Left atrial strain parameters via TTE**			
**LASr, %**	15.56 ± 5.08	27.61 ± 5.23	<0.0001
**LAScd, %**	−7.94 ± 4.29	−14.18 ± 5.64	<0.0001
**LASct, %**	−8.10 ± 3.08	−13.21 ± 3.21	<0.0001

Values are mean ± standard deviation or median [25th–75th percentile, *n* (%)], unless otherwise indicated.

LAVI, left atrial volume index; LVEDV, left ventricular end diastolic volume; LVESV, left ventricular end systolic volume; LVEF, left ventricular ejection fraction; MVA, mitral valve area; MVMG, mitral valve mean gradient; TAPSE, tricuspid annular plane systolic excursion; LAAEV, left atrial appendage emptying velocity; LASEC, left atrial spontaneous echo contrast; LASr, left atrial reservoir strain; LAScd, left atrial conduit strain; LASct, left atrial contractile strain.

### Coagulation profile assessment

**Figure 2:**
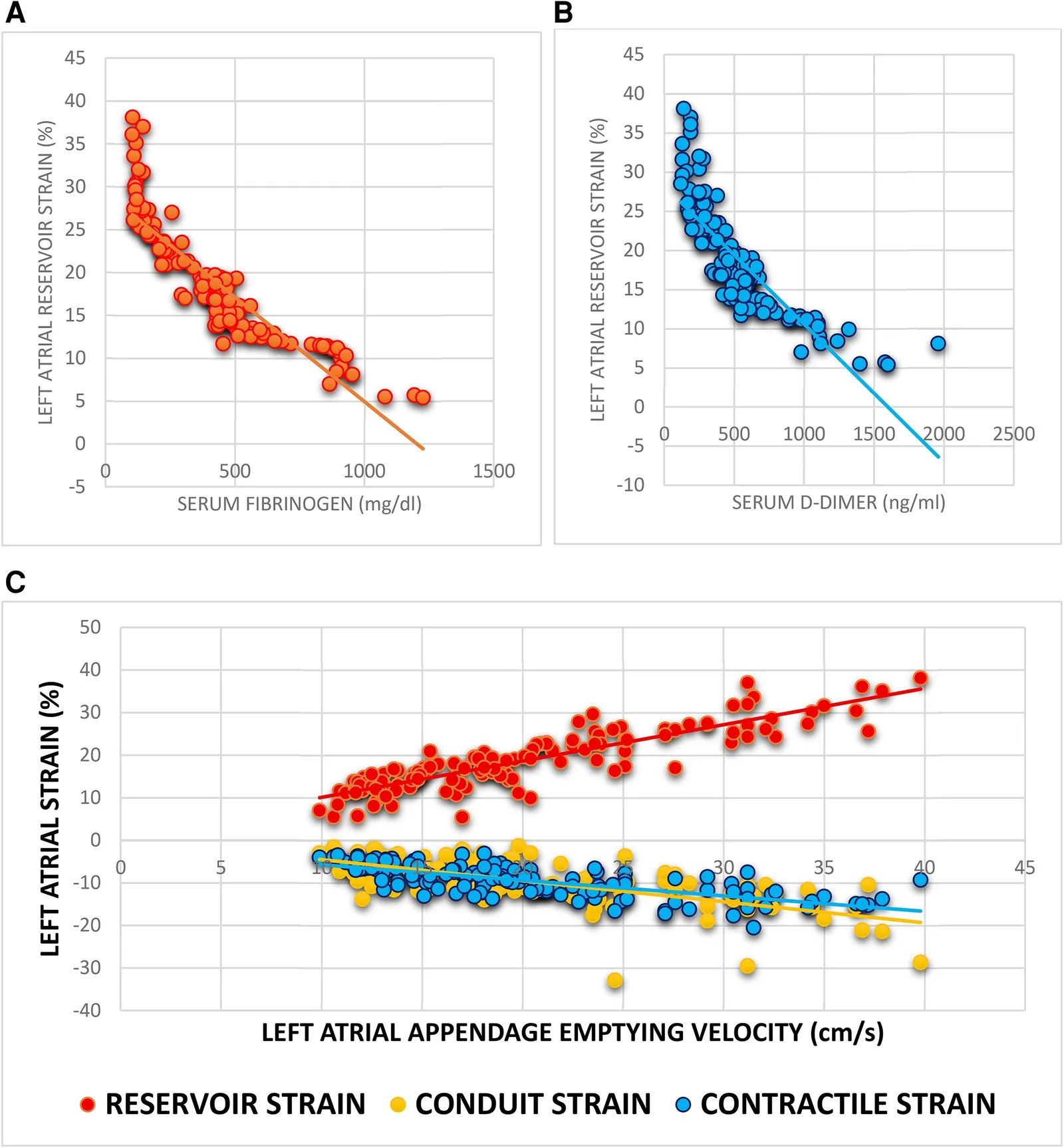
Scatter plot showing the correlation of left atrial reservoir strain with serum fibrinogen, D-dimer, and LAAEV. Each point represents an individual patient, illustrating an inverse association between LASr and both fibrinogen, D-dimer levels, conduit, and contractile strain.

Serum fibrinogen and D-dimer levels were increased with LAA inactivity as a marker of stasis, and there was a negative correlation as the two variables move in opposite directions. Hence, both serum fibrinogen and D-dimer had a strong inverse correlation with appendage velocities and LA strain (*Figure 2A* and *B*; Supplementary material online, *Table S1*).

**Figure 3:**
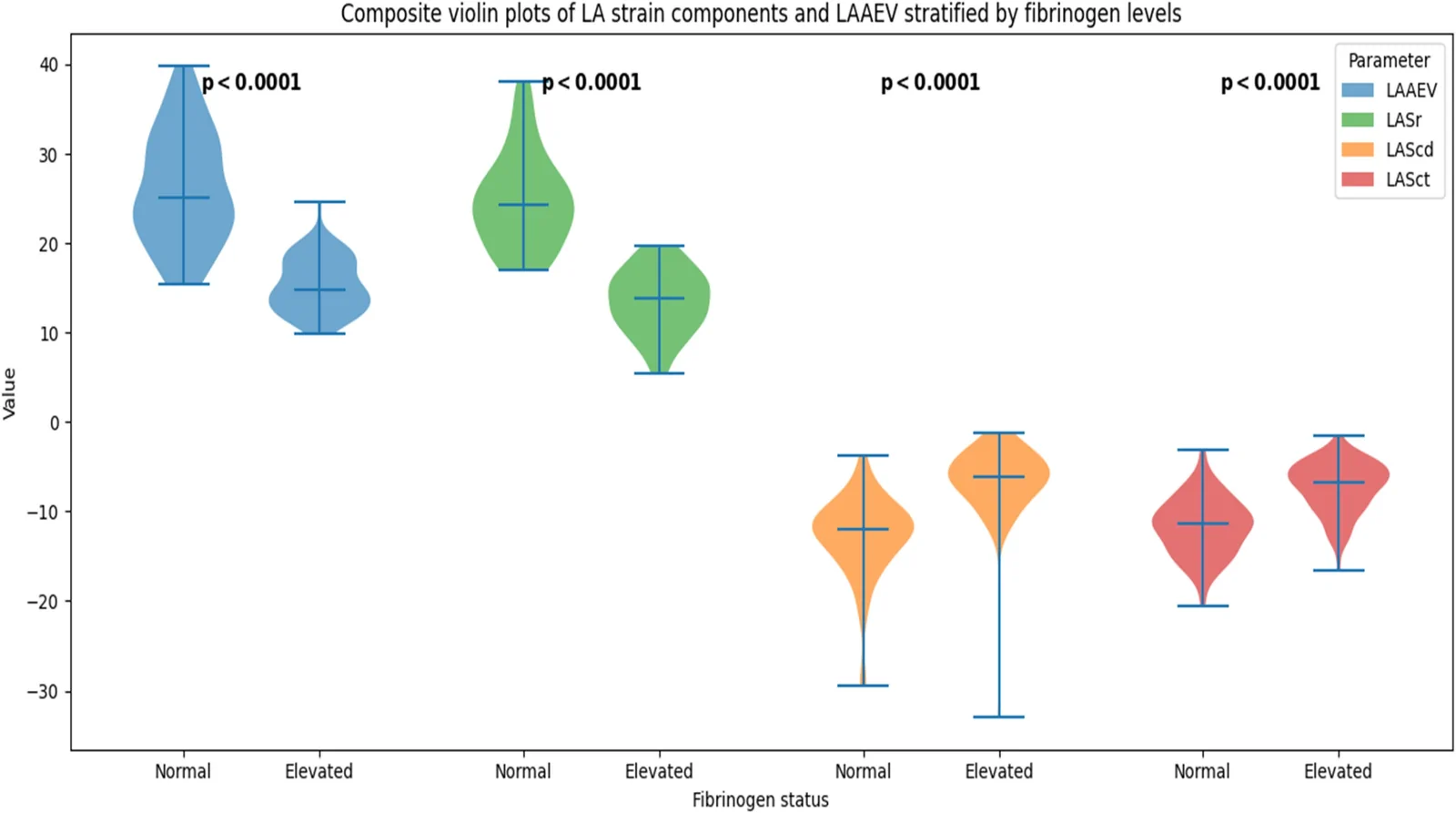
Composite violin plots demonstrating left atrial functional parameters and LAAEV stratified by fibrinogen status. Composite violin plots showing LAAEV, LASr, LAScd, and LASct in patients with normal and elevated fibrinogen levels. Colour-coded violins represent individual parameters. LAVI, left atrial volume indices; LASr, left atrial reservoir strain; LAScd, left atrial conduit strain; LASct, left atrial contractile strain; LVEDV, left ventricular end diastolic volume; MVA 2D, mitral valve area by planimetry; MVA PHT, mitral valve area by pressure half time; MVMG, mitral valve mean gradient; MVPG, mitral valve peak gradient.

More than half of the cohort had raised fibrinogen levels (57.2%), while approximately half had elevated D-dimer (50.7%), with nearly one-half demonstrating elevation of both markers (47.8%), indicating a substantial burden of hypercoagulability. Patients with elevated fibrinogen showed significantly impaired LASr and LAScd and LAAEV. Among patients with inactive LAA (*n* = 108), elevated fibrinogen (>400 mg/dL) was present in 79 (73.1%) patients and elevated D-dimer (>500 ng/mL) in 70 (64.8%), and both markers were elevated in 66 (61.1%) patients (*Figure 3*; Supplementary material online, *Tables S2* and *S3*).

### Subgroup analysis of patients with left atrial appendage thrombus

Among 138 patients with severe rheumatic MS in SR, eight (5.8%) were found to have LAA thrombus on TEE, underscoring that clinically silent thrombus formation can occur even in the absence of AF.

Patients with LAA thrombus had a non-significant larger mitral valve area (1.12 ± 0.20 cm^2^), comparable to patients without thrombus (0.97 ± 0.22 cm^2^, *P* = 0.084), and higher *trans*-mitral gradients, and increased LAVI, but statistically, it was not significant (see Supplementary material online, *Table S4*). Mean LAAEV (14.88 ± 2.72 cm/s) was significantly reduced compared with the non-thrombus group in the overall cohort (20.19 ± 7.16 cm/s, *P* = 0.00037). In the inactive group only, though the mean LAAEV was lower compared to the patients with no LAA thrombus (*n* = 100), statistically, it was not significant (14.88 ± 2.72 cm/s vs. 16.93 ± 3.94, *P* = 0.078). Left atrial reservoir strain was markedly reduced in the thrombus group (9.56 ± 1.08% vs. 16.15 ± 4.95%, *P* < 0.00001). Similarly, both LAScd (−4.75 ± 1.04% vs. −8.20 ± 4.35%, *P* < 0.00001) and LASct (−4.79 ± 1.41% vs. −8.37 ± 3.03%, *P* < 0.00001) were significantly lower in patients with thrombus, indicating more severe global atrial mechanical dysfunction. The coagulation parameters revealed a clear hypercoagulable milieu in the thrombus subset, with higher fibrinogen, though it was not statistically significant with no thrombus cohort in the inactive LAA group (567.12 ± 88.36 mg/dL vs. 510.82 ± 236.92 mg/dL, *P* = 0.1691). The D-dimer was also higher in patients in the thrombus subgroup (768.75 ± 198.96 ng/mL vs. 624.60 ± 319.98 ng/mL, *P* = 0.0912); however, the difference was not statistically significant (see Supplementary material online, *Table S4*).

### Correlation of left atrial appendage inactivity with left atrial strain imaging

There was a strong positive correlation of LASr with LAAEV (*r* = 0.857 (*P* < 0.0001). LAScd and LASct also had a strong inverse correlation with LAAEV (*Figure 2C*; Supplementary material online, *Table S1*).

### Univariate predictors of left atrial appendage inactivity

**Figure 4:**
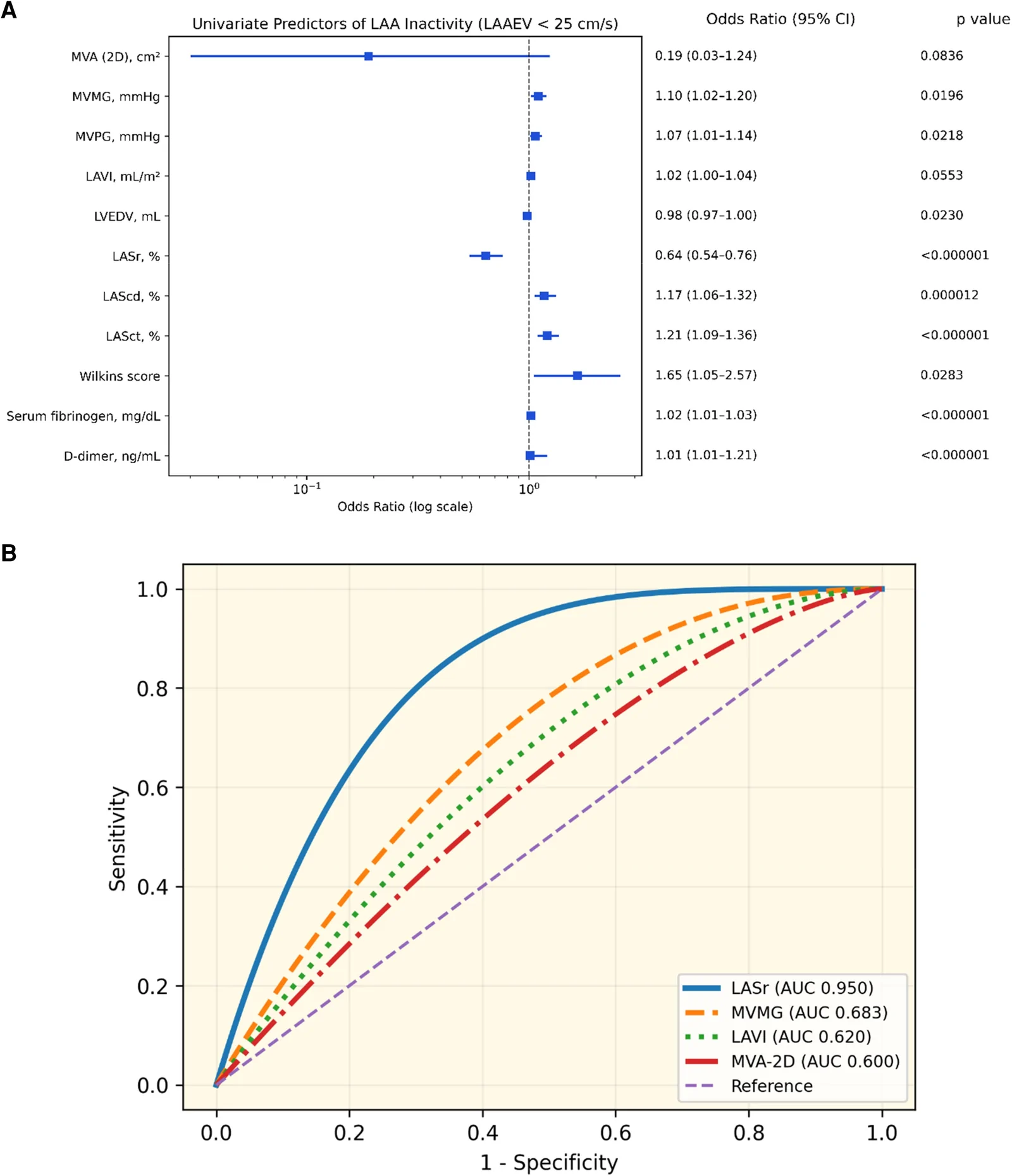
Forest plot showing univariate analysis of echocardiographic parameters to predict LAA inactivity (*A*). Comparison of ROC curve showing the discriminative ability of the LASr (AUC-0.950), MVA-2D (AUC-0.600), MVMG (AUC-0.683), and LAVI (AUC-0.620) for LAA inactivity (*B*). AUC, area under curve; LAAEV, left atrial appendage emptying velocity; LAScd, left atrial conduit strain; LASct, left atrial contractile strain; LASr, left atrial reservoir strain; ROC, receiver operating characteristics.

On univariate analysis, higher *trans*-mitral gradients, mitral valve mean gradient [MVMG; odds ratio (OR): 1.10, 95% CI: 1.02–1.20; *P* = 0.0196], mitral valve peak gradient (MVPG; OR: 1.07, 95% CI: 1.01–1.14; *P* = 0.0218), lower left ventricular end diastolic volume (LVEDV; OR: 0.98, 95% CI: 0.97–1.00, *P* = 0.023), and high Wilkins score (OR: 1.65, 95% CI: 1.05–2.57, *P* = 0.0283) were significantly associated with LAA inactivity (*Figure 4A*; Supplementary material online, *Table S5*).

In multivariable logistic regression, all three LA strain parameters—LASr, LAScd, and LASct—were significantly associated with LAA inactivity. Among them, LASr emerged as the strongest independent echocardiographic predictor (OR: 0.61 per 1% increase; 95% CI: 0.50–0.75; *P* < 0.0001), followed by LAScd (OR: 1.27; 95% CI: 1.12–1.44; *P* < 0.0001) and LASct (OR: 1.65; 95% CI: 1.32–2.01; *P* < 0.0001). The model incorporating LASr achieved a good overall fit (Hosmer–Lemeshow *P* = 0.944) and diagnostic accuracy of 90.6%. Each 1% increase in LASr was associated with a 39% reduction in the odds of LAA inactivity (*Table 3*).

**Table 3:** Multivariate analysis showing the independent predictors of left atrial appendage inactivity

Variable	Reference	Model 1		Model 2		Model 3	
		Odds ratio [95% CI]	*P*-value	Odds ratio [95% CI]	*P*-value	Odds ratio [95% CI]	*P*-value
MVA PHT, cm^2^	Per unit	0.54 [0.01–24.14]	0.756	0.23 [0.01–3.81]	0.120	0.71 [0.04–14.39]	0.822
MVMG, mmHg	Per unit	1.03 [0.90–1.19]	0.670	1.02 [0.94–1.12]	0.545	1.07 [0.97–1.17]	0.154
LVEDV, mL	Per unit	0.98 [0.96–1.00]	0.102	0.99 [0.97–1.01]	0.191	0.98 [0.96–1.00]	0.064
Wilkin’s score	Per unit	1.89 [0.83–4.31]	0.154	1.62 [0.91–2.88]	0.093	1.52 [0.84–2.72]	0.165
LAVI, mL/m^2^	Per unit	0.98 [0.95–1.01]	0.247	1.01 [0.98–1.03]	0.598	1.00 [0.98–1.02]	0.683
MVA-2D, cm^2^	Per unit	0.77 [0.02–25.92]	0.886	0.66 [0.05–9.10]	0.758	1.81 [0.11–30.25]	0.680
Reservoir strain, %	Per unit	0.61 [0.50–0.75]	<0.001	–	–	–	–
Conduit strain, %	Per unit	–	–	1.27 [1.12–1.44]	<0.001	–	–
Contractile strain, %	Per unit	–	–	–	–	1.65 [1.33–2.01]	<0.001
Goodness of fit	Overall accuracy	90.6%		82.6%		84.8%	
	Hosmer–Lemeshow test	0.944		0.668		0.080	
	−2log value	56.87		102.54		88.39	

AUC, area under curve; MVA, mitral valve area; MVMG, mitral valve mean gradient; MVPG, mitral valve peak gradient; LVEDV, left ventricular end diastolic volume.

On ROC analysis, LASr yielded an AUC of 0.950 (95% CI: 0.911–0.989), with a threshold ≤ 22.95% showing sensitivity of 93.5% and specificity of 90.0%. For LAScd, a cut-off of ≥−9.7% provided a sensitivity of 69.4% and specificity of 86.7%, while LASct ≥ −11.35% yielded a sensitivity of 82.4% and specificity of 73.3% (*Table 4* and *Figure 4B*). All patients (*n* = 46) in the lowest LASr tertile (≤14.63%) had LAAEV < 25 cm/s, indicating LAA inactivity (see Supplementary material online, *Table S6*).

**Table 4:** Receiver operating characteristic analysis showing optimal cut-off values of left atrial strain for predicting left atrial appendage inactivity

Variables	AUC	AUC 95% CI	*P*-value	ROC cut-off value	Sensitivity %	Specificity %
**LASr, %**	0.950	0.911–0.989	<0.001	≤22.95	93.5	90.0
**LAScd, %**	0.842	0.763–0.920	<0.001	≥−9.7	69.4	86.7
**LASct, %**	0.868	0.800–0.936	<0.001	≥−11.35	82.4	73.3

AUC, area under curve; ROC, receiver operating characteristic; LASr, left atrial reservoir strain; LAScd, left atrial conduit strain; LASct, left atrial contractile strain.

Comparative ROC analysis demonstrated that LASr had superior discriminatory performance with AUC = 0.950 (95% CI: 0.911–0.989) compared with MVMG (AUC = 0.683; 95% CI: 0.573–0.793), LAVI (AUC = 0.620; 95% CI: 0.502–0.737), and MVA-2D (AUC = 0.600; 95% CI: 0.488–0.711), highlighting the incremental value of functional atrial assessment over conventional structural and haemodynamic parameters for predicting LAA inactivity (*Figure 4B*).

### Sensitivity analysis

To address the potential impact of a high event rate on model performance, a sensitivity analysis was performed using a more stringent definition of LAA inactivity (LAAEV ≤ 18 cm/s), so that event rate is nearly equal 68/138 (49.3%) and 70/138 (50.7%). Notably, LASr demonstrated the highest discriminative ability with AUC of 0.924 (95% CI: 0.88–0.97) (see Supplementary material online, *Table S7*).

In multivariable modelling, the baseline model incorporating conventional echocardiographic parameters (LAVI, MVA-2D, MVMG, MV-PHT, LVEDV, and Wilkins score) demonstrated a −2 log-likelihood of 123.77. The addition of LASr significantly improved model fit, reducing the −2 log-likelihood to 56.87 (Δχ^2^ = 36.90, *P* < 0.001), indicating substantial incremental value. Similarly, inclusion of conduit and contractile strain improved model performance, with −2 log-likelihood values of 102.54 and 88.39, respectively. Among these, reservoir strain provided the greatest incremental diagnostic value.

### Intraclass correlation coefficient analysis

The inter-observer variability for LA strain variables ranged from 0.89 to 0.98 which seems to be excellent (see Supplementary material online, *Table S8*).

## Discussion

Rheumatic heart disease remains a significant public health concern in developing countries, contributing substantially to cardiovascular morbidity and mortality.^1,26,27^ While thromboembolic risk in MS has traditionally been attributed to AF, emerging evidence highlights that patients in SR may also harbour LA and LAA dysfunction, predisposing them to thrombus formation.^2–5^

This study demonstrates that LAA mechanical dysfunction is highly prevalent in severe rheumatic MS, even in the absence of AF. Among 138 patients in SR, 78% exhibited LAA inactivity, indicating a substantial burden of atrial contractile dysfunction in this group. This finding aligns with prior work by Mukhopadhyay *et al*., who reported a 73% prevalence of LAA inactivity, although LA strain analysis was not included in their study.^7^

Importantly, biochemical markers of hypercoagulability—fibrinogen and D-dimer—were significantly elevated in patients with LAA inactivity, reflecting an underlying prothrombotic state. Over half of the cohort had elevated fibrinogen (57.2%), nearly half had elevated D-dimer (50.7%), and 47.8% had both markers elevated. In the LAA-inactive group, these proportions increased further: 73.1% had elevated fibrinogen, 64.8% elevated D-dimer, and 61.1% both. These findings align with previous studies, which linked rheumatic MS and LA dysfunction to systemic activation of coagulation markers, suggesting a coupled mechanical and biochemical profile of thrombotic risk.^8,12,28^

All three LA strain components—reservoir (LASr), conduit (LAScd), and contractile (LASct)—were significantly reduced in patients with LAA inactivity. Among them, LASr had the strongest correlation with LAAEV (*r* = 0.857, *P* < 0.0001), outperforming LAScd (*r* = −0.670) and LASct (*r* = −0.721). The ROC analysis identified LASr ≤ 22.95% as the optimal cut-off for predicting LAA inactivity, with a sensitivity of 93.5%, specificity of 90.0%, and an AUC of 0.950, indicating excellent diagnostic performance. These results position LASr as a reliable surrogate marker of LAA dysfunction and a potential non-invasive alternative to TEE in selected patients.

Several studies in non-valvular AF and cryptogenic stroke populations have linked impaired LA strain with LAA stasis and thrombus formation. Karabay *et al*. reported that LASr < 11.8% was associated with thrombus in stroke patients in SR.^19^ Zhu *et al*. found LASr < 13% predicted LAA stasis in non-valvular AF,^21^ and Wang *et al*. showed peak LA strain predicted LAA dysfunction with AUC > 0.81.^29^ Providência *et al*. demonstrated moderate correlations between LA strain rate and LAA emptying velocities in non-valvular AF.^30^ Compared to these studies, the stronger correlation observed in our cohort may reflect the homogeneity of pathophysiology in rheumatic MS vs. the heterogeneous mechanisms in non-valvular AF. The higher LASr cut-off in our study (≤22.95%) compared to non-valvular AF populations likely reflects distinct pathophysiology. In rheumatic MS, LA remodelling is driven primarily by chronic pressure overload in a younger demographic with preserved ventricular function, unlike older AF patients with advanced fibrosis and electrical remodelling. These differences underscore the need for context-specific strain thresholds and interpretation.

The LAA thrombus was detected in eight patients (5.8%) despite SR, a prevalence consistent with prior studies.^2–5^ These patients exhibited significantly impaired LA strain across all phases and lower LAAEV, indicating profound atrial mechanical dysfunction. Elevated fibrinogen and D-dimer levels in this group further confirmed the presence of a hypercoagulable milieu, although not reaching statistical significance due to small sample size. These findings reinforce that atrial rhythm alone is insufficient to exclude thrombotic risk; atrial mechanical assessment adds critical prognostic value.

Strain-based stratification by LASr tertiles revealed a stepwise worsening of atrial function, thrombotic risk markers, and LAAEV. All patients in the lowest LASr tertile (≤14.63%) had LAA inactivity, dense smoke, and elevated fibrinogen and D-dimer levels, whereas the highest tertile (>20.7%) was associated with preserved LAA function and favourable profiles. These findings highlight the potential of LASr not only as a diagnostic tool but also for clinical risk stratification and therapeutic decision-making—possibly identifying patients in SR who might benefit from anticoagulation.

Notably, traditional anatomical parameters—mitral valve area, Wilkins score, and transmitral gradients—did not independently predict LAA inactivity, indicating that mechanical assessment is more sensitive to thromboembolic risk than structural severity alone. This shift towards functional risk assessment aligns with emerging trends in atrial disease management.

The recently proposed CHA_2_DS_2_-VASc-LA score aims to improve stroke risk stratification in elderly rheumatic AF patients.^31^ However, in younger rheumatic populations lacking age-related risk factors, incorporating a dynamic functional marker like LA strain may provide superior prognostic value. We propose the concept of a CHA_2_DS_2_-VASc-LA strain score, integrating LA strain as a physiologically meaningful extension of current risk models.

### Clinical implications and potential applications

- Strain imaging provides a sensitive, quantitative echocardiographic marker for LAA mechanical dysfunction, helping uncover hidden thromboembolic risk in patients with severe rheumatic MS who remain in SR.
- Combining atrial deformation parameters with biochemical markers in severe rheumatic MS in SR may guide risk stratification and early initiation of anticoagulation in patients with inactive LAA.

### Limitations

First, this was a single-centre observational study with a limited sample size, which may affect the generalizability of the findings. Second, despite using 24-h Holter monitoring, paroxysmal AF could not be completely ruled out. Third, the lack of long-term follow-up prevented assessment of actual thromboembolic outcomes. Finally, we did not evaluate the effect of interventions such as balloon mitral valvuloplasty on LA strain or LAA function.

## Conclusions

This study is the first to demonstrate that LASr, measured via transthoracic strain imaging, independently predicts LAA inactivity in patients with severe rheumatic MS in SR. The LA strain parameters correlate strongly with appendage function, systemic hypercoagulability, and the presence of LAA thrombus. Reservoir strain (LASr) in particular showed high diagnostic accuracy and may serve as a non-invasive surrogate for TEE-derived LAA assessment. Strain-based stratification can identify patients at the highest thromboembolic risk despite being in SR, offering a new paradigm in anticoagulation decision-making. Incorporating LA strain into routine evaluation may bridge the current gap in thromboembolic risk assessment and improve individualized care in rheumatic MS.

## Supplementary Material

oeag130_Supplementary_Data

## Data Availability

The datasets generated and/or analysed during the current study are not publicly available to maintain patient confidentiality but are available from the corresponding author on reasonable request and after the agreement of all the co-authors.
