# Supplementary material for: Prediction of left atrial appendage dysfunction by atrial strain imaging in patients with rheumatic mitral stenosis in sinus rhythm
Source: Eur Heart J Open. 2026 Aug 20;6(5):oeag130. doi: 10.1093/ehjopen/oeag130 (PMC13574500; doi:10.1093/ehjopen/oeag130)
Supplement: Supplementary file 1 [file oeag130_supplementary_data.docx]

SUPPLEMENTAL MATERIAL

Contents

Supplemental Table 1. Correlation of LAAEV with coagulation and LA strain parameters (Spearman’s correlation)………………………………………………………………………….33

**Supplemental Table 2. Serum fibrinogen and D-dimer levels in severe rheumatic MS in sinus rhythm…………………………………………………………………………….…34**

[Supplemental Table 3. Echocardiographic and left atrial functional parameters stratified by fibrinogen and D-dimer levels……………………………………………...](#_Toc218718978)35

[Supplemental Table 4. Structural, functional, and coagulation parameters in rheumatic MS patients with and without LAA thrombus in sinus rhythm………………………..](#_Toc218718979)36

**Supplemental Table 5. Univariate Predictors of LAA Inactivity (LAAEV < 25 cm/s)..37**

**Supplemental Table 6.** **Echocardiographic and coagulation parameters in three tertiles as per reservoir strain……………………………………………………………………..38**

**Supplemental Table 7.** **Sensitivity analysis based on LAAEV ≤18 cm/s………………. …...…………………………………………………………………………………………40**

**Supplemental Table 8.** **Intraclass Correlation Coefficient Analysis……………………. …...…………………………………………………………………………………………41**

**Supplemental Table 1**: Correlation of LAAEV with coagulation and LA strain parameters (Spearman’s correlation)

| Variable | LAAEV(Cm/s) |
| --- | --- |
| Fibrinogen | -0.821 (p<0.001) |
| D-dimer | -0.786 (P<0.001) |
| LASr | 0.857 (p<0.0001) |
| LAScd | -0.670 (p<0.0001) |
| LASct | -0.721 (p<.0001) |

LASr-left atrial reservoir strain; LAScd-left atrial conduit strain; LASct-left atrial contractile strain.

**Supplemental** **Table 2:** Serum fibrinogen and D-dimer levels in severe rheumatic MS in sinus rhythm.

| **Group** | **Overall cohort n=138 (%)** | **Inactive LAA cohort**  **n=108 (%)** |
| --- | --- | --- |
| Elevated Fibrinogen | 79 (57.2%) | 79 (73.1%) |
| Elevated D-dimer | 70 (50.7%) | 70 (64.8%) |
| Both Elevated | 66 (47.8%) | 66 (61.1%) |

**Supplemental** **Table 3:** Echocardiographic and left atrial functional parameters stratified by fibrinogen and D-dimer levels.

| **Parameter** | **Normal Fibrinogen (<400 mg/dL) Mean ± SD** | **Elevated Fibrinogen (≥400 mg/dL) Mean ± SD** | **p value** | **Normal D-dimer**  **(<500 ng/mL) Mean ± SD** | **Elevated D-dimer**  **(≥500 ng/mL) Mean ± SD** | **p value** |
| --- | --- | --- | --- | --- | --- | --- |
| **LASr (%)** | 24.73 ± 5.24 | 13.43 ± 3.57 | <0.0001 | 23.64 ± 5.69 | 13.04 ± 3.53 | <0.0001 |
| **LAScd (%)** | -12.88 ± 4.59 | -6.63 ± 4.03 | <0.0001 | -12.27 ± 4.63 | -6.42 ± 4.15 | <0.0001 |
| **LASct (%)** | -11.73± 3.28 | -7.33±2.89 | <0.0001 | -11.46±3.29 | -7.21 ± 2.95 | <0.0001 |
| **LAAEV (cm/s)** | 26.07 ± 6.24 | 15.28 ± 3.06 | <0.0001 | 24.83 ± 6.69 | 15.09 ± 3.07 | <0.0001 |

Values are mean ± standard deviation. LASr-left atrial reservoir strain; LAScd-left atrial conduit strain; LASct-left atrial contractile strain; LAAEV, left atrial appendage emptying velocity

**Supplemental** **Table 4:** Structural, functional, and coagulation parameters in rheumatic MS patients with and without LAA thrombus in sinus rhythm.

| Parameters | Inactive LAA with thrombus (n=8) | Inactive LAA without thrombus (n=100) | p value |
| --- | --- | --- | --- |
| Age (yrs) | 38.12 ± 6.38 | 34.87 ± 8.12 | 0.2079 |
| MVA (2D) (cm²) | 1.12 ± 0.20 | 0.97 ± 0.22 | 0.0844 |
| MVMG (mmHg) | 16.12 ± 7.77 | 13.31 ± 6.29 | 0.3481 |
| MVPG (mmHg) | 25.75 ± 9.16 | 22.03 ± 8.86 | 0.2998 |
| LAVI (ml/m²) | 75.66 ± 19.67 | 62.17 ± 27.58 | 0.1037 |
| LAAEV (cm/s) | 14.88 ± 2.72 | 16.93 ± 3.94 | 0.078 |
| LASr (%) | 9.56 ± 1.08 | 16.15 ± 4.95 | <0.00001 |
| LAScd (%) | -4.75 ± 1.04 | -8.20 ± 4.35 | <0.00001 |
| LASct (%) | -4.79 ± 1.41 | -8.37 ± 3.03 | <0.00001 |
| Fibrinogen (mg/dL) | 567.12 ± 88.36 | 510.82 ± 236.92 | 0.1691 |
| D-dimer (ng/mL) | 768.75 ± 198.96 | 624.60 ± 319.98 | 0.0912 |

Values are mean ± standard deviation, unless otherwise indicated.

MVA, mitral valve area; MVMG, mitral valve mean gradient; MVPG, mitral valve peak gradient; LAVI, left atrial volume index; LAAEV, left atrial appendage emptying velocity; LASr-left atrial reservoir strain; LAScd-left atrial conduit strain; LASct-left atrial contractile strain.

**Supplemental Table 5:** Univariate Predictors of LAA Inactivity (LAAEV < 25 cm/s)

| Parameter | Odds Ratio (95 % CI) | p-value |
| --- | --- | --- |
| MVA (2D), cm² | 0.19 (0.03 – 1.24) | 0.0836 |
| MVA (PHT), cm^2^ | 0.11 (0.02-0.78) | 0.0270 |
| MVMG, mmHg | 1.10 (1.02 – 1.20) | 0.0196 |
| MVPG, mmHg | 1.07 (1.01 – 1.14) | 0.0218 |
| LAVI, ml/m² | 1.02 (1.00 – 1.04) | 0.0553 |
| LVEDV, mL | 0.98 (0.97-1.00) | 0.0230 |
| LASr, % | 0.64 (0.54 – 0.76) | < 0.000001 |
| LAScd, % | 1.17 (1.06 – 1.32) | <0.000001 |
| LASct, % | 1.21 (1.09 – 1.36) | < 0.000001 |
| Wilkins score | 1.65 (1.05-2.57) | 0.0283 |
| Serum fibrinogen (mg/dL) | 1.02 (1.01 – 1.027) | < 0.000001 |
| D-dimer (ng/mL) | 1.01 (1.01 – 1.21) | < 0.000001 |

LASr-left atrial reservoir strain; LAScd-left atrial conduit strain; LASct-left atrial contractile strain; LAVI- left atrial volume index; LVEDV, left ventricular end diastolic volume; MVA, mitral valve area; MVMG, mitral valve mean gradient; MVPG, mitral valve peak gradient

**Supplemental Table 6**: Echocardiographic and coagulation parameters in three tertiles as per reservoir strain

| LASr | Tertile 1  ≤14.63%  (n=46) | Tertile 2  14.63 to 20.7%  (n=46) | Tertile 3  >20.7%  (n=46) | Total | P value |
| --- | --- | --- | --- | --- | --- |
| LAScd (%) | -5.72 ± 2.49 | -8.85 ± 4.52 | -13.88 ± 4.48 | -9.48 ± 5.17 | <0.0001^*^ |
| LASct (%) | -6.11 ± 1.86 | -9.05 ± 2.84 | -12.63 ± 2.88 | -6.11 ± 1.86 | <0.0001^*^ |
|  |  |  |  |  |  |
| LAAEV (cm/s) | ≤14.63%  (n=46) | 14.63 to 20.7%  (n=46) | >20.7%  (n=46) | Total | P value |
| Inactive {<25 cm/s} | 46 (100.00%) | 44 (95.65%) | 18 (39.13%) | 108 (78.26%) | <0.0001^†^  <0.0001^†^ |
| Active {≥25 cm/s} | 0 (0.00%) | 2 (4.35%) | 28 (60.87%) | 30 (21.74%) |  |
| LAAEV (cm/s) | 14.37 ± 2.83 | 17.93 ± 3.19 | 27.84 ± 5.66 | 20.05 ± 7.02 | <0.0001^*^ |
|  |  |  |  |  |  |
| COAGULATION PARAMETERS | ≤14.63%  (n=46) | 14.63 to 20.7%  (n=46) | >20.7%  (n=46) | Total | P value |
| Serum Fibrinogen | 700.80 ± 212.58 | 437.54 ± 56.08 | 177.93 ± 60.74 | 438.76 ± 250.97 | <0.0001^*^ |
| Serum D-dimer | 904.78 ± 339.61 | 538.96 ± 122.35 | 253.48 ± 82.68 | 565.74 ± 341.50 | <0.0001^*^ |
| ^†^ Chi square test, ^*^Kruskal Wallis test | |  |  |  |  |

Values are mean ± standard deviation. LAAEV, left atrial appendage emptying velocity; LAAFV, left atrial appendage filling velocity; LASr-left atrial reservoir strain; LAScd-left atrial conduit strain; LASct-left atrial contractile strain.

**Reservoir strain tertiles analysis**

The study cohort was further divided into three tertiles based on LASr (Tertile-1; ≤14.63%, Tertile-2; 14.63-20.7%, Tertile-3; >20.7%) to show a graded deterioration in the atrial mechanics. A clear gradient was observed across tertiles, with significant stepwise improvement in both LAScd and LASct strain from lowest to highest LASr groups (p < 0.0001). LAAEV also showed marked differences among the three tertiles. In the lowest LASr tertile, all 46 patients (100%) had LAAEV <25 cm/s, indicating complete LAA inactivity. In contrast, only 18 of 46 (39.13%) patients in the highest tertile had LAAEV <25 cm/s, with 28 (60.87%) exhibiting preserved LAA function (p<0.0001). Mean LAAEV progressively increased across tertiles (LAAEV: 14.37 cm/s in the lowest vs. 27.84 cm/s in the highest tertile, supporting strong coupling between global atrial and appendage mechanics. Hypercoagulability markers showed a similar gradient: fibrinogen levels were 700.80, 437.54, and 177.93 mg/dL, and D-dimer were 904.78, 538.96, and 253.48 ng/mL across increasing LASr tertiles (p < 0.0001). (**Supplementary Table 6**)

**Supplemental Table 7:** Sensitivity analysis based on LAAEV ≤18 cm/s

| Variable | Reference | Model-1 | | Model-2 | | | Model -3 | | |
| --- | --- | --- | --- | --- | --- | --- | --- | --- | --- |
|  |  | Odds ratio  [95% CI] | P-value | Odds ratio  [95% CI] | P-value | Odds ratio  [95% CI] | | P-value |  |
| MVA PHT cm^2^ | Per unit | 0.02 [0.001–0.59] | 0.024 | 0.04 [0.003–0.593] | 0.019 | 0.13 [0.01–1.53] | | 0.104 |  |
| MVMG, mmHg | Per unit | 0.97 [0.89 – 1.06] | 0.541 | 1.00 [0.93–1.07] | 0.969 | 1.02 [0.95 – 1.09] | | 0.557 |  |
| LVEDV, ml | Per unit | 0.99 [0.97–1.01] | 0.284 | 0.98 [0.96–1.00] | 0.087 | 0.99 [0.97–1.00] | | 0.135 |  |
| WILKIN SCORE | Per unit | 0.82 [0.49–1.36] | 0.441 | 1.08 [0.69–1.69] | 0.747 | 0.95 [0.6–1.49] | | 0.836 |  |
| LAVI | Per unit | 1.00[0.98-1.02] | 0.962 | 1.00[0.98-1.02] | 0.963 | 1.01 [0.99-1.02] | | 0.390 |  |
| MVA2D | Per unit | 6.83[0.28-167.50] | 0.886 | 2.22[0.17-29.03] | 0.545 | 1.28[0.12-13.74] | | 0.839 |  |
| RESERVOIR STRAIN, % | Per unit | 0.63 [0.53 – 0.74] | <0.001 | – | – | – | | – |  |
| CONDUIT STRAIN, % | Per unit | – | – | 1.51[1.30–1.77] | <0.001 | – | | – |  |
| CONTRACTILE STRAIN, % | Per unit | – | – | – | – | 1.51 [1.29–1.76] | | <0.001 |  |
| GOODNESS OF FIT | Overall accuracy | 85.5% |  | 80.4% |  | 74.6% | |  |  |
|  | Hosmer-Lemeshow test  -2log value  AUC ROC | 0.278  9.81  0.924[0.88-0.97] |  | 0.664  6.03  0.868[0.806-0.931] |  | 0.297  9.56  0.849[0.787-0.910] | |  |  |

AUC, area under curve; MVA, mitral valve area; MVMG, mitral valve mean gradient; MVPG, mitral valve peak gradient; LVEDV, left ventricular end diastolic volume

# **Supplemental Table 8:** Intraclass Correlation Coefficient Analysis

| **Variable** | **ICC** | **95% CI** |
| --- | --- | --- |
| LAAEV | 0.944 | 0.834-0.981 |
| Reservoirs strain | 0.943 | 0.828-0.981 |
| Conduit Strain | 0.978 | 0.936-0.993 |
| Contractile Strain | 0.891 | 0.673-0.963 |
